# Black Crowberry (*Empetrum nigrum* L.) Flavonoids and Their Health Promoting Activity

**DOI:** 10.3390/molecules21121685

**Published:** 2016-12-07

**Authors:** Tunde Jurikova, Jiri Mlcek, Sona Skrovankova, Stefan Balla, Jiri Sochor, Mojmir Baron, Daniela Sumczynski

**Affiliations:** 1 Institute for Teacher Training, Faculty of Central European Studies, Constantine the Philosopher University in Nitra, Drazovska 4, SK-949 74 Nitra, Slovakia; sballa@ukf.sk; 2 Department of Food Analysis and Chemistry, Faculty of Technology, Tomas Bata University in Zlin, nam. T. G. Masaryka 5555, CZ-760 01 Zlin, Czech Republic; mlcek@ft.utb.cz (J.M.); skrovankova@ft.utb.cz (S.S.); sumczynski@ft.utb.cz (D.S.); 3 Department of Viticulture and Enology, Faculty of Horticulture, Mendel University in Brno, Valticka 337, CZ-691 44 Lednice, Czech Republic; sochor.jirik@seznam.cz (J.S.); MojmirBaron@seznam.cz (M.B.)

**Keywords:** flavonoids, polyphenols, phenolic acids, anthocyanins, antioxidant activity

## Abstract

Nowadays, much research attention is focused on underutilized berry crops due to the high antioxidant activity of fruits. Black crowberry (*Empetrum nigrum* L.) represents an important source of flavonols (quercetin, rutin, myricetin, naringenin, naringin, morin, and kaempferol) and anthocyanins. The fruit components could be utilised as natural colourants or as a part of functional foods and, because of the high antioxidant activity, the berries of black crowberry can be used in the treatment of diseases accompanied with inflammation, or as an effective antibacterial and antifungal remedy. Moreover, the reduction of lipid accumulation and total cholesterol as well as an improvement of postprandial hyperglycaemia have been proven. This review summarizes for the first time the main antioxidants (flavonoids) of black crowberry fruits, with a focus on their health promoting activity.

## 1. Introduction

Recently, the attention of researchers has been focused on lesser-known and underutilized species of edible berries, such as honeyberry, lingonberry, rowanberry, black crowberry, and others with respect to their health benefits [1,2,3,4]. Wild species of fruits such as *Empetrum nigrum*, *Vaccinium ulinosum*, *Vaccinium vitis ideae*, *Aronia melanocarpa*, *Vaccinium oxycoccus*, and *Sorbus aucuparia* display higher antioxidant activity in comparison with well-known and distributed berry crops (strawberry, raspberry) [5]. Moreover, black crowberry (Empetraceae) is on the top list of families with the highest content of antioxidants [6]. It is also a reason why crowberry is one of the leading representatives on the list of “prospective super foods” [7].

Black crowberry includes two forms—*E. nigrum* (crowberry or black crowberry) and its tetraploid subspecies *E. nigrum* ssp. *hermaphroditum* [8]. Black crowberry is a commonly occurring wild berry in the northern hemisphere—Europe, Eurasia, and Canada [9,10], the fresh crowberry fruit is a part of the traditional diet in North American tribal communities [7], but the fresh fruits are sometimes classified as inedible in botanical literature. This is due to the high content of tannins that are responsible for raw fruits’ taste. They are slightly acidic, bitter, and astringent and taste better after freezing. Therefore, fruit products such as juices and jams or mixtures with other berries or wines are preferred for consumption [11]. Another utilisation of crowberry fruit is its usage as a medicinal plant in folk medicine; e.g., as anti-inflammatory agents for the treatment of cystitis, nephritis, and urethritis, a practice in use in Korea [12]. The health promoting activity of the fruit is given by the high content of polyphenol compounds [13]—especially flavonols (quercetin, kaempferol, and myricetin) and anthocyanins—that have content and structure similar to blueberry fruit [14,15]. Despite the possibilities of fruit application, the crowberry is not commonly utilized in commercial products [11]. As mentioned above, fresh black crowberry fruits have the potential for utilisation in the future as a part of functional foods [16] and they are also an effective component for the cosmetic and pharmaceutical industries [17]. Powdered dried fruits of *E. nigrum* could also be used as ingredients in functional foods [18].

A summary of the main antioxidants—flavonoids—of black crowberry fruit together with the utilization of health promoting compounds has not yet been published. Moreover, this review brings a comparison of these bioactive compounds with components of other underutilized plant species. This could therefore help to clarify the position of black crowberry fruit with respect to anthocyanin content, as these are the most important flavonoids in the fruit.

## 2. Overview of Main Polyphenols with Attention to Flavonoids

Polyphenols (especially flavonoids) represent the main group of bioactive compounds of *Empetrum nigrum* fruit, and contribute to the high antioxidant and health promoting activities of plants [1,4]. 

### 2.1. Polyphenols

Polyphenols are the most significant bioactive compounds in black crowberry fruit. The total polyphenol content (TPC) of black crowberry fruit represents 4.3 ± 0.09 mg GAE/g DW (gallic acid equivalent) in fresh fruit and 7.51 ± 0.17 mg GAE/g DW in dried fruit [18], although the total content of phenolic compounds in the leaves of black crowberry is higher than in the fruit. Park et al. examined the TPC of black crowberry fruit originating from Korea by Folin-Ciocalteu method, resulting in the content of 39 ± 2 mg/100 g FW (fresh weight) of fruit [12]. 

The fruit of black crowberry also represents a valuable source of anthocyanins (460 mg/100 g FW fruit) [19]. The content of flavonoids (as the predominant group of polyphenols) as catechin equivalent (CE)/g DW of fresh and dried *Empetrum* fruits was 2.46 ± 0.01 and 3.94 ± 0.106 mg CE/g DW, respectively [18]. Crowberries are further rich in flavonols [13,20,21]. Another group of polyphenols presented in black crowberry fruit are phenolic acids. Ogawa et al. [14] identified caffeic, gallic, and protocatechuic acids in black crowberry samples. Laaksonen et al. [11] detected the presence of the prevailing hydroxycinnamic acid and its conjugates—*p-*coumaric acid and caffeic acid in black crowberry fruit from northern Finland using high performance liquid chromatography (HPLC) method with DAD (diode array) detector. The total sum of phenolic acids reported by Dudone [22], analysed by ultra-high performance liquid chromatography tandem mass spectrometry (UHPLC-MS/MS), was 12.1 ± 0.80 mg/100 g FW. The experimental results showed *p*-coumaric acid as the major phenolic acid (8.33 ± 0.63 mg/100 g FW), and other forms of acid were present in lower amounts (*m*-coumaric 1.33 ± 0.09 mg/100 g FW; coumaric acid glucoside 1.14 ± 0.03 mg/100 g FW). Furthermore, they also detected *p*-hydroxybenzoic acid (0.47 ± 0.03 mg/100 g FW), ferulic acid (0.21 ± 0.01 mg/100 g FW), caffeic acid (0.18 ± 0.01 mg/100 g FW), and protocatechuic acid (0.19 ± 0.03 mg/100 g FW) [22].

Most research concerning black crowberry polyphenol composition is performed in relation to another lesser known fruit species. Dudonne et al. [22] provided a comparative study of the polyphenol composition for twelve native Canadian berries: Saskatoon berry, alpine bearberry, chokeberry, black crowberry, honeysuckle, chokecherry, cloudberry, elderberry, lowbush blueberry, alpine blueberry, lingonberry, and highbush cranberry. The TPC of black crowberry was analysed by Folin-Ciocalteu measurement (454 ± 3.77 mg/100 g FW). The fruit had the third highest value after highbush cranberry (762 ± 4.21 mg/100 g FW) and black chokeberry (603 ± 14.9 mg/100 g FW). The measured value for black crowberry fruit is lower in comparison to Bakowska-Barczak’s [23] study of common Canadian berries (690 mg/100 g FW). 

However, the measurement of polyphenols and their composition could be varied depending on the climate. The content of polyphenols and their composition was also investigated in fruits ripened under tundra conditions. The concentration of stilbens increased under light grazing and warming. On the other hand, under the mentioned conditions, some parameters decreased, such as the concentration of flavonols and condensed tannins, with no change in total phenolic content. Under heavy grazing, a weak but consistent decrease among the different phenolic compound groups was evident, resulting in a decrease in total phenolics [24,25,26]. The study of Kellogg et al. [25] confirmed significant variation in anthocyanins (0.01–4.39 mg/100 g FW) and proanthocyanidins (0.74–6.25 mg/g FW), depending on the site of cultivation. All research studies documented the exceptional position of black crowberry fruit in terms of polyphenol content, especially flavonoids and their significant changes under different climatic and site conditions.

### 2.2. Flavonoids

Flavonoids represent the predominant group of phenolic compounds. Flavonols, flavan-3-ols, and anthocyanins are the most important flavonoids in crowberry fruit. A summary of *Empetrum nigrum* flavonoids is given in Table 1.

#### 2.2.1. Flavonols

Flavonols are the most important group of flavonoids in black crowberry fruit. Ogawa et al. [14] identified quercetin, rutin, myricetin, naringenin, naringin, catechin, morin, and kaemferol as the main flavonols of *Empetrum nigrum* fruits. A more detailed spectrum of black crowberry flavonoids was provided by HPLC-DAD analyses by Laakson et al. [11]. According to the authors, 35 flavonols were identified in the fruit of black chokeberry and were presented as glycosides, galactosides, glucosides, arabinosides, and xylosides of myricetin and quercetin; moreover laricitrin, isorhamnetin, and syringetin were also detected. Quercetin-3-*O*-galactoside and quercetin-3-*O*-glucoside were determined as main flavonols.

According to the results of the flavonoids of crowberry fruit originating from Korea [12], determined by HPLC method, the content of quercetin and kaempferol was 23 ± 1.5 mg/100 g FW or 21.5 ± 0.5 mg/100 g FW, respectively. Dudonne et al. [22] provided detailed UHPLC-MS/MS analyses of flavonols (37.2 ± 2.89 mg/100 g FW), with quercetin as the predominant compound. In fruit samples, quercetin was present as quercetin-3-glucoside (13.6 ± 0.99 mg/100 g FW), quercetin-3-arabinose (5.71 ± 0.41 mg/100 g FW), quercetin-3-xyloside (4.17 ± 0.36 mg/100 g FW), quercetin-3-galactoside (2.12 ± 0.29 mg/100 g FW), quercetin-3-rhamnoside (0.45 ± 0.04 mg/100 g FW), and free quercetin (0.48 ± 0.07 mg/100 g FW). Kaempferol represented only a small part of the sample (0.72 ± 0.3 mg/100 g FW), and was present in the glucoside and galactoside forms.

The total content of crowberry flavonols—quercetin, myricetin, and kaempferol—reached up to higher levels in comparison with commonly consumed fruits or vegetables such as onion, kale, and broccoli [20]. Mlcek et al. [27] reported quercetin content above 20 mg/100 g FW in some berries and fruits, including black crowberry, lingonberry, cranberry, blueberry, blackcurrant, blue grapes, rosebud, apple, and apricot.

#### 2.2.2. Flavan-3-ols

In black crowberry fruit, flavan-3-ols represent the second most abundant group of flavonoids. Among flavan-3-ols in black crowberry fruit, epicatechin predominated, in quantities of 7.09 ± 0.41 mg/100 g FW. That is a higher concentration compared to the amount found in Saskatoon berry, chokeberry, or honeysuckle [1,3,22]. Proanthocyanidins (0.74–6.25 mg/100 g FW) were present as the A-type proanthocyanidin polymers analysed by HPLC and LC-MS (liquid chromatography tandem mass spectrometry) [25]. 

#### 2.2.3. Anthocyanins as the Main Antioxidants of Crowberry Fruit

Black crowberry is a unique and exceptional source of anthocyanins. Crowberry fruit has high levels of anthocyanin pigments in the skin, which have potential as natural food colourants and food additives [18].

The first attempt to quantify anthocyanins comes from Linko et al. and Käppa et al. [15,19]. The fruits of *Empetrum nigrum* subsp. *hermaphroditum* (Hagerup Böcker) were extracted by ethanol and acetic acid and purified by extraction on Ionex. Separation and analyses of aglycones were provided by HPLC method, and glycoside units were analysed by GLC-MS (gas liquid chromatography tamdem mass spectrometry). Researchers identified 12 acetylated monosides: glucosides, galactosides, arabinosides of delphinidin, petunidin, cyanidin, peonidine, and malvidine. The three main anthocyanins of *Empetrum nigrum* fruit were galactosides of cyanidin, delphinidin, and malvidin, which formed about 60% of total anthocyanins. More detailed identification and quantification of individual anthocyanidins was provided by Ogawa et al., using 80% methanol with 0.5% acetic acid for extraction [14]. They identified thirteen kinds of anthocyanins (in accordance with the results of experiments provided by Laaksonen et al. [28] and Heyman et al. [29]), with the highest concentration in the press residue of juice. 

Generally, anthocyanins are present in the form of glycosides in the majority of berry crops [28]. The predominant anthocyanins are represented by galactosides—cyanidin-3-galactoside and delphinidin-3-galactoside—in amounts more than 24% of the total anthocyanin content (8.04 and 8.62 mg/g extract, respectively) [14,19,30]. In recent studies by Kellogg et al. and Koskela et al. [25,31], there were 15 anthocyanins identified in crowberry juice. The total content of anthocyanins represented 127.6 ± 1.4 mg/100 mL of fresh juice (quantified as cyanidin-3-glucoside equivalents). Thus, the conclusion is that the 3-galactosides of malvidin, delphinidin, cyanidin, petunidin, and peonidin were the most frequent, followed by 3-arabinosides and 3-glucosides of the same anthocyanidins [32].

There are interesting comparative studies of anthocyanin quantification and identification among berry crops. The fruits of black crowberry together with bilberry [9] present an important source of galactosides of delphinidines—the main representative of the berry crop. The total anthocyanin content of black crowberry (41.8 mg/100 g extract) is very similar to bilberry (38.8 mg/100g), which represents one of the richest sources of anthocyanins [13,14,33]. The results of the experiment of Ogawa et al. [14] showed that the total amount of anthocyanins in black crowberry fruit was the highest among nine analysed berry crops (2.5–38.8 mg/g of extract). On the basis of detailed analyses of the anthocyanins of Scandinavian berry crops, in black crowberry, petunidin-3-galactoside is present in relatively high amounts in comparison with other berry crops [21].

Anthocyanins in black crowberry, chokeberry, and blackcurrant juices are often analysed by high performance liquid chromatography coupled with mass spectrometry. The experimental results showed that crowberry juice had a more variable anthocyanin profile compared to chokeberry and blackcurrant juices. In fruit juices, the total anthocyanin content varied from 44.8 to 128 mg/100 mL, with the highest value in crowberry juice. Generally, cyanidin-3-galactoside was the most abundant in lingonberry, alpine bearberry, Saskatoon berry, chokeberry, and black crowberry [32]. One of the most comprehensive research works of Bae et al. [30] compared the anthocyanin content of black crowberry with four major berry species—Korean black raspberry, mulberry, strawberry, and blueberry. The experimental results proved that black crowberry displayed the highest anthocyanin content. In their study, Dudonne et al. [22] investigated the anthocyanin content and anthocyanidin spectrum of twelve native Canadian berries. Anthocyanin contents reached values from 2 to 503 mg/100 g FW, with the highest content in black crowberry.

Anthocyanins are highly unstable and very susceptible to degradation. The stability of crowberry anthocyanins was examined by Kallio et al. [34] in relation to storage temperature, the presence of ascorbic acid, and Fe^3+^ and Al^3+^ ions. Authors found out that the half-life (t_1/2_) of pure untreated crowberry juice was 4–6 weeks. When 90% of the oxygen was removed, the stability was improved by a factor of 3 to 4. Glucosides and galactosides had a longer degradation half-life than arabinosides. The presence of Fe^3+^ and Al^3+^ ions increased the stability of anthocyanins, but significantly decreased the colour shade of the juice. In the dark-coloured crowberry juice, the total anthocyanin content constitutes about 45%–50% of the fruit juice. Due to the relatively bland taste of the juice it may have future use as a natural food colourant [14,35]. 

Hellstrom et al. [32] compared the stability of chokeberry, blackcurrant, and crowberry juices. The results of the experiment showed that the optimal stability for chokeberry juice is t_1/2_ for total anthocyanins = 6.7 weeks at 21 °C; 23.8 weeks at 9 °C; and 32.5 weeks at 4 °C) whilst in blackcurrant it is t_1/2_ = 3.0 weeks at 21 °C; 11.5 at 9 °C; and 20.3 weeks at 4 °C, respectively, and in crowberry juice is t_1/2_ = 2.2 weeks at 21 °C; 7.3 at 9 °C; and 12.3 weeks at 4 °C, respectively. 

Crowberry fruit can be utilised as a natural colour enhancer; a summary of the main anthocyanins in the fruit of black crowberry is depicted in Table 2.

## 3. Antioxidant Activity of Crowberry Fruits

The evaluation of the antioxidant activity of fruits is a way of expressing the biological activity of fruit that should be coupled with the study of the major biologically active substances [36,37,38,39] to provide comprehensive insight. In the case of black crowberry, fruit flavonoids predominate.

The extract of crowberry fruits restores the activity of cellular antioxidant enzymes, such as superoxide dismutase, catalase, glutathione peroxidase, and heme oxygenase-1 [40], and therefore it effectively suppresses hydrogen peroxide and ultraviolet B (UVB) induced cell damage [40,41]. Park et al. [12] studied 1,1-diphenyl-2-pycryl-hydrazyl (DPPH) scavenging activity, anti-low-density lipoprotein (LDL) oxidation activity, and protective effects of H_2_O_2_-induced cytotoxicity using fruit extracts of black crowberry from Korea. The fruit extract displayed a strong antioxidative effect in the free radical scavenging and superoxide dismutase (SOD)-like enzyme. The black crowberry fruit extract also inhibited lipid peroxidation in human LDL, detected as a reduction in malondialdehyde production [28,38].

The total antioxidant activity in the crowberry (*Empetrum hermaphroditum*) (9.63 mmol/L) was reported to be higher than in blackcurrant (5.49 mmol/L), blueberry (7.57 mmol/L), strawberry (7.01 mmol/L), and raspberry (4.01 mmol/100 mL) [42]. All radical scavenging methods (DPPH, 2,2′-azino-bis(3-ethylbenzothiazoline-6-sulfonic acid (ABTS) and ferric reducing ability of plasma (FRAP) tests) displayed the strongest antioxidant activity of crowberry, compared to other berry crops such as blackberry, bilberry, blackcurrant, blueberry, mulberry, raspberry, cranberry, and red currant. Antioxidant potencies were indicated as a percentage of radical quenching activity (DPPH and ABTS) and mg Trolox equivalent/mL. These results suggest that the crowberry is one of the most powerful antioxidant berries [14]. 

Hakkinen et al. [10] found that the extracts of crowberry, cloudberry, whortleberry, aronia, rowanberry, and cranberry possess high antioxidant activity. Among 92 edible plant materials studied by Kakhonen et al. [21] (berries, fruits, vegetables, herbs, cereals, tree materials, plant sprouts, and seeds), berry crops—especially aronia and crowberry showed exceptionally high antioxidant activity and TPC (GAE > 20 mg/g).

Polyphenolic compounds are a major group of secondary metabolites that mostly contribute to the antioxidant activity of crowberry. Black crowberry fruit showed a good correlation of free radical scavenging activity with TPC and TFC (total flavonoid content) (*r*^2^ = 0.921 and 0.890, respectively, with significance at *p* < 0.001) [17,30].

## 4. Health Promoting Activity of Black Crowberry Fruit

Berries of black crowberry—in relation to their high flavonoid content—are reported to have potential health-promoting effects, such as antioxidative, anti-inflammatory, and anticarcinogenic effects [4]. 

In folk medicine, the fruits of black crowberry are utilized for the treatment of epileptic and paralytic states. Crowberry extract is a part of an homeopathic drug for the treatment of epilepsy in Russia (Empetrin). It also displays astringent and diuretic properties [43]. 

Crowberry has been used in folk medicine to treat inflammatory diseases [44]. Hyun et al. [17] tried to clarify the anti-inflammatory activity of the crowberry aerial part extract. RAW 264.7 cells were stimulated with LPS (lysophosphatidylcholine) in the presence of the crude methanolic extract and its fractions at various concentrations, followed by determination of NO production in the LPS-stimulated cells. The results proved that aerial part extract of crowberry suppresses the secretion of pro-inflammatory mediator from LPS-stimulated cells. The crude MeOH extract and EtOAc fraction at 200 μg/mL inhibited LPS-induced NO production by 82% and 60%, respectively.

The most-studied activity in black crowberry fruit is antimicrobial (AM) and antifungal activity, firstly published by and Maatsuura et al. [45]. The antimicrobial activity of berries is given by multiple mechanisms of bioactive compounds and their synergies, such as weak organic acids, phenolic acids, and tannins and their complexes in different chemical forms; therefore, the antimicrobial effects of chemically complex compounds must be critically analysed [46].

Rauha et al. [47] studied the phenolic spectrum of berry extracts (cloudberry, raspberry, and crowberry) using the agar diffusion method, and showed their antibacterial effects against several bacterial strains. Liisa et al. [46] examined the antimicrobial activity of phenolic extracts of 12 Nordic fruit species, including black crowberry fruit. The results of the experiments showed strong inhibition against *Bacillus cereus* and weak inhibition against *Campylobacter jejuni*. Another similar study by Rauha et al. [47] proved that the methanolic extract of crowberry flavonoids has slight antimicrobial activity (1–3 mm and more inhibition zone of methanol) against *Staphylococcus aureus*, *Staphylococcus epidermis*, and *Escherichia coli*, moderate antimicrobial activity (3–4 mm) against *Bacillus subtilis*, and clear antimicrobial activity (4–10 mm) against *Micrococcus luteus*. The juice of black crowberry is also effective against pneumococcal infections. 

Paudel et al. [48] studied the antimicrobial activity against *Staphylococcus aureus*, *Candida albicans*, and *E. coli* within 51 species of higher plants collected from the Oymyakon region of the Republic of Sakha (Yakutia) and Russia. The results were evaluated according to the size of the inhibition zone. The findings showed that only two species of higher plants—*Empetrum nigrum* and *Cassiope tetragona*—displayed very strong AM activity against *S. aureus* (inhibition zone: ≥20 mm) and moderate AM activity against *C. albicans* (inhibition zone: 10 mm).

Huttunen et al. [49] compared the inhibitory activity of cranberry, bilberry, and crowberry (*E. nigrum* and *E. hermaphroditum* L.) juice fractions against *Streptococcus pneumoniae.* Human bronchial cells (Calu-3) were used as an adhesion model. All studied berry juices showed remarkable antiadhesive and AM activity. Pneumococcal growth was totally inhibited at a concentration of ~86 mg/g polyphenolic fraction of *Empetrum nigrum* juice. The strongest effect was displayed by the cranberry juice fraction. Gordien et al. [50] studied different extracts from plants, lichens, and fungal endophytes of Scottish provenance for activity against *Mycobacterium aurum* and *M. tuberculosis* H(37)Rv. The results of the experiment showed that the highest activity against *M. tuberculosis* was noticed for extracts of *C. arbuscula*, *E. nigrum*, and *J. communis* roots, *Calluna vulgaris* aerial parts, and *Myrica* gale roots and stems (93% to 99% inhibition at 100 μg/mL). The fruit of black crowberry also contains 2′-methoxy-4′-hydroxy-α- and β–dihydrochalcone with antimicrobial activity [51]. Anti-mycobacterial activity of the plant extract was given by the presence of two chalcone derivatives that exhibited selective anti-mycobacterial activity (IC_50_ values of 23.8 and 32.8 μM, respectively) in comparison to mammalian (HEK 293) cells (IC_50_ values of 109 and 249 μM, respectively), which was confirmed by a study by Li et al. [52].

The anticancer activity of ethyl acetate extract of *Empetrum nigrum* fruit is based on protective actions against UVB radiation in human HaCaT keratinocytes (aneuploid immortal keratinocyte cell line from adult human skin) [41]. Several authors have described more mechanisms of *E. nigrum*’s activity such as absorbing UVB radiation and scavenging UVB-induced intracellular reactive oxygen species (ROS) in HaCaT keratinocytes. Moreover, *E. nigrum* protected HaCaT keratinocytes in the level of cellular components (e.g., against peroxidation of lipids, modification of proteins, and breakage of DNA strands) following UVB irradiation and against UVB-induced apoptotic cell death [41]. In addition, *E. nigrum* recovered cell viability by inhibiting apoptosis after cells were exposed to radiation. *E. nigrum* treatment also reduced γ-radiation-induced Bax proteins (apoptosis regulator), and caspase 9 and 3 expression in irradiated cells. The mechanism of the anti-apoptotic effect of *E. nigrum* is given by the inhibition of mitogen-activated protein kinase-4 (MKK4/SEK1)-c-Jun N-terminal kinase (JNK) cascades induced by γ-radiation [40].

The black crowberry can be utilised in food processing as well. Wines made from a mixture of crowberry and blackcurrants have slightly higher antioxidant activity compared to red grape wines [53]. The studies of Eiro et al. and Rein et al. [54,55,56] proved that the addition of crowberry juice improved the colour quality of blackcurrant wine, significantly enhancing the colour. The colour of enhanced wine was about 35% more intense in comparison with plain black currant juice. The colour intensity of blackcurrant wine enriched with crowberry juice decreased by about 60% after six months of storage. This is an indication of copigmentation reactions taking place during fermentation and storage of the blackcurrant wines.

Black crowberry fruit also seems to be an effective antidiabetic agent in fortified preparations of functional drinks. Torronen et al. [16] studied the effect of fortification of blackcurrant juice by black crowberry powdered fruit extract and its effect on polyphenol composition, urinary and plasma phenolic metabolites, and postprandial glycaemic response in healthy subjects. The TPC was doubled after the juice fortification process, from 159 up to 293 mg/100 mL. Urinary metabolites such as dihydroxybenzoic acid sulphate and dihydroxyphenylacetic acid sulphate increased after intake, and were present in higher levels after intake of the fortified juice. Compared to pure blackcurrant juice, the fortified juice elicited slightly attenuated and sustained plasma glucose and insulin responses. The mixture of crowberry and blackcurrant improved postprandial glycemic response of a 36 g sugar dose, due to increased bioavailability of polyphenols [57]. The ethanolic fraction of the aerial part of the black crowberry plant displays high-level α-glucosidase inhibitory activity. This indicates that crowberry can potentially reduce postprandial hyperglycemia by delaying carbohydrate digestion, and can be utilised as an alternative antidiabetic drug [17].

Proanthocyanidin-enriched extracts of alpine blueberry and black crowberry were found to reduce lipid accumulation in mouse adipocytes, in a positive correlation with total Proanthocyanidin content [25].

Human studies of the antioxidant effect of black crowberry fruit were provided by Park et al. [12]. They evaluated the effect of crowberry on antioxidative activities, homocysteine level, and lipid profile of subjects. Fifty-one healthy volunteers consumed 2 g of powdered crowberry every day for four weeks. The research results proved that regular crowberry consumption caused a significant increase in total antioxidant status and superoxide dismutase. Moreover, the level of total cholesterol and low-density lipoprotein significantly decreased. The differences in levels of antioxidant markers and lipid profiles taken before and after crowberry intake were considerable. After crowberry intake, the levels of homocysteine (Hcy), catalase, triglyceride, and LDL significantly decreased. 

Crowberries are good dietary sources of natural antioxidants with a dominant composition of flavonoids, α-glucosidase inhibitory, and anti-inflammatory components, suggesting that crowberry consumption can be a potential natural way to improve human health [17].

## 5. Conclusions

There is a current preference for the use of lesser-known fruits like black crowberry in fruit production. This is because this species contains great amounts of biologically active substances, especially flavonoids (flavonols and anthocyanins), that display high antioxidant activity. For this reason, crowberry has a promising potential for future utilization as an alternative herbal drug displaying antidiabetic and antibiotic effect. Moreover, this species displays anti–inflammatory and anticancer activity, and has a positive effect on lipid metabolism. Crowberry extract can also find use as a nutraceutical component or for functional foods.

## Figures and Tables

**Table 1:** Overview of main black crowberry (*Empetrum nigrum*) flavonoids (total content).

Detected Phenolic Compounds	Literature Source
**Flavonols**	
Quercetin	23 ± 1.5–37.2 ± 2.89 mg/100 g FW Park et al. [12], Dudonne et al. [22]
Kaempferol	21.5 ± 0.5 mg/100 g FW Park et al. [12]
**Flavan-3-ols**	
Epicatechin	7.09 ± 0.41 mg/100 g FW Dudonne et al. [22]
Proanthocyanidins	0.74–6.25 mg/g FW Kellogg et al. [25]
Anthocyanins	41.8 mg/g extract Koponen et al. [33] 503 mg/100 g FW Dudonne et al. [22]

**Table 2:** Anthocyanins in *Empetrum nigrum* fruits.

Detected Anthocyanins	Literature Source
Cyanidin-3-galactoside	Kellogg et al. [25], Koskela et al. [31], Ogawa et al. [14], Helstrom et al. [32], Heyman et al. [29], Dudonne et al. [22]
Cyanidin-3-glucoside	Ogawa et al. [14], Kellogg et al. [25], Helstrom et al. [32], Heyman et al. [29]
Cyanidin-3-arabinoside	Ogawa et al. [14], Kellogg et al. [25], Koskela et al. [31], Helstrom et al. [32], Heyman et al. [29], Dudonne et al. [22]
Delphinidin-3-galactoside	Kappa et al. [19], Ogawa et al. [14], Kellogg et al. [25], Koskela et al. [31], Helstrom et al. [32], Heyman et al. [29], Dudonne et al. [22]
Delphinidin-3-glucoside	Kappa et al. [19], Ogawa et al. [14], Kellogg et al. [25], Koskela et al. [31], Helstrom et al. [32], Heyman et al. [29]
Delphinidin-3-arabinoside	Kappa et al. [19], Ogawa et al. [14], Kellogg et al. [25], Koskela et al. [31], Helstrom et al. [32], Heyman et al. [29], Dudonne et al. [22]
Peonidin-3-galactoside	Ogawa et al. [14], Kellogg et al. [25], Koskela et al. [31], Helstrom et al. [32], Heyman et al. [29], Dudonne et al. [22]
Peonidin-3-arabinoside	Kellogg et al. [25], Koskela et al. [31], Helstrom et al. [32], Heyman et al. [29], Dudonne et al. [22]
Peonidin-3-glucoside	Kellogg et al. [25], Koskela et al. [31], Helstrom et al. [32], Heyman et al. [29]
Petunidin-3-galactoside	Kappa et al. [19], Ogawa et al. [14], Kellogg et al. [25], Koskela et al. [31], Helstrom et al. [32], Heyman et al. [29], Dudonne et al. [22]
Petunidin-3-glucoside	Kappa et al. [19], Ogawa et al. [14], Kellogg et al. [25], Koskela et al. [31], Helstrom et al. [32], Heyman et al. [29]
Petunidin-3-arabinoside	Kappa et al. [19], Ogawa et al. [14], Kellogg et al. [25], Koskela et al. [31], Helstrom et al. [32], Heyman et al. [29]
Malvidin-3-arabinoside	Kappa et al. [19], Ogawa et al. [14], Kellogg et al. [25], Koskela et al. [31], Helstrom et al. [32], Heyman et al. [29], Dudonne et al. [22]
Malvidin-3-galactoside	Kappa et al. [19], Kellogg et al. [25], Koskela et al. [31], Helstrom et al. [32], Heyman et al. [29], Dudonne et al. [22]
Malvidin-3-glucoside	Kappa et al. [19], Kellogg et al. [25], Koskela et al. [31], Helstrom et al. [32], Heyman et al. [29]
